# Supplementary material for: pH- and sodium-induced changes in a sodium/proton antiporter
Source: eLife. 2014 Jan 28;3:e01412. doi: 10.7554/eLife.01412 (PMC3900740; doi:10.7554/eLife.01412)
Supplement: Supplementary file 1. — Lower table legend: a Calculated from the program AVRGAMPS: mean weighted squared distance of the phase values from the averaged value (90° = random) b Calculated from the program FOMSTATS: averaged phase values from the symmetry-constrained target values of 0° or 180° (45° = random). a,b Reflections with IQ ≤ 7 Å were included. DOI: http://dx.doi.org/10.7554/eLife.01412.013 [file elife01412s001.docx]

**Supplementary file 1:** Electron crystallographic data.

| Condition | | pH4 -NaCl | | pH4 + 20 mM NaCl | | pH4 + 100 mM NaCl | | pH4 + 150 mM NaCl | |
| --- | --- | --- | --- | --- | --- | --- | --- | --- | --- |
| Unit cell dimensions | | a= 81.5 Å, b= 103.3 Å, γ=90° | | a= 81.8 Å, b= 103.8 Å, γ=90° | | a= 81 Å, b= 103.8 Å, γ=90° | | a= 80.8 Å, b= 104.9 Å, γ=90° | |
| Space group | | *p*22_1_2_1_ | | | | | | | |
| Resolution | | 6 Å | | | | | | | |
| No. of images | | 11 | | 10 | | 9 | | 20 | |
| Range of defocus | | 270-560 nm | | 318-911 nm | | 563-823 nm | | 362-704 nm | |
| Total no. of reflections^a^ | | 2236 | | 1898 | | 1614 | | 3817 | |
| No. of unique reflections^b^ | | 199 | | 199 | | 193 | | 201 | |
| Overall phase residual to 6 Å  (AVRGAMPS, random= 90°)^a^ | | 30.5° | | 33.1° | | 27.9° | | 30.1° | |
| Overall phase residual to 6 Å  (sym. constraint, random= 45°)^b^ | | 19° | | 24.5° | | 26° | | 16.8° | |
| Statistics  (FOMSTATS,  random= 45°) | Res (Å)  19.2  13.5  11.0  9.5  8.5  7.8  7.2  6.7  6.3  6.0 | Reflections  23  20  21  18  21  18  20  20  17  20 | PhRes (°)  16.3  10.3  9.7  21.2  17.2  16.3  16.2  29.8  27.8  27.1 | Reflections  23  20  22  17  21  21  19  18  16  22 | PhRes (°)  20.0  18.5  15.0  15.8  18.0  21.4  28.7  33.5  27.9  46.9 | Reflections  22  21  20  16  22  16  22  18  17  19 | PhRes (°)  17.9  18.2  18.7  25.3  26.4  20.7  31.4  39.5  21.3  41.5 | Reflections  22  20  23  19  20  18  21  18  18  22 | PhRes (°)  22.0  14.4  10.5  9.2  17.9  16.2  15.7  13.5  23.5  24.3 |

^a^ Calculated from the program AVRGAMPS: mean weighted squared distance of the phase values from the averaged value (90° = random) ^b^ Calculated from the program FOMSTATS: averaged phase values from the symmetry-constrained target values of 0° or 180° (45° = random). ^a,b^ Reflections with IQ ≤ 7 Å were included.

**Supplementary file 1: Electron crystallographic data. (continued)**

| Condition | | pH4 + 250 mM NaCl | | pH4 + 500 mM NaCl | | pH4 + 100 mM LiCl | | pH4 + 250 mM LiCl | |
| --- | --- | --- | --- | --- | --- | --- | --- | --- | --- |
| Unit cell dimensions | | a= 80.5 Å, b= 106.4 Å, γ=90° | | a= 79.3 Å, b= 105.9 Å, γ=90° | | a= 81.1 Å, b= 104.6 Å, γ=90° | | a= 80.5 Å, b= 105.3 Å, γ=90° | |
| Space group | | *p*22_1_2_1_ | | | | | | | |
| Resolution | | 6 Å | | | | | | | |
| No. of images | | 16 | | 7 | | 8 | | 11 | |
| Range of defocus | | 290-652 nm | | 353-658 nm | | 450-827 nm | | 206-761 nm | |
| Total no. of reflections^a^ | | 3434 | | 1482 | | 1457 | | 2182 | |
| No. of unique reflections^b^ | | 202 | | 189 | | 197 | | 199 | |
| Overall phase residual to 6 Å  (AVRGAMPS, random= 90°)^a^ | | 26.1° | | 26.1° | | 29.3° | | 30.9° | |
| Overall phase residual to 6 Å  (sym. constraint, random= 45°)^b^ | | 16.2° | | 17.2° | | 20.9° | | 18.9° | |
| Statistics  (FOMSTATS,  random= 45°) | Res (Å)  19.2  13.5  11.0  9.5  8.5  7.8  7.2  6.7  6.3  6.0 | Reflections  22  20  23  19  19  21  19  17  21  21 | PhRes (°)  16.3  13.4  19.8  11.3  10.8  18.3  10.6  14.5  25.0  19.4 | Reflections  20  19  22  20  18  18  20  16  17  19 | PhRes (°)  21.8  12.2  15.9  13.6  6.3  14.4  16.3  16.2  26.4  29.6 | Reflections  21  20  21  18  21  19  20  19  17  21 | PhRes (°)  10.1  11.8  13.3  13.2  11.2  16.6  32.0  30.6  33.6  38.7 | Reflections  21  20  23  19  19  20  20  16  19  22 | PhRes (°)  11.4  12.9  26.6  17.9  15.0  16.8  21.0  14.8  22.1  28.2 |

^a^ Calculated from the program AVRGAMPS: mean weighted squared distance of the phase values from the averaged value (90° = random) ^b^ Calculated from the program FOMSTATS: averaged phase values from the symmetry-constrained target values of 0° or 180° (45° = random). ^a,b^ Reflections with IQ ≤ 7 Å were included.

**Supplementary file 1: Electron crystallographic data. (continued)**

| Condition | | pH4 + 100 mM KCl | | pH4 + 25 mM MgCl_2_ | | pH4 - NaCl  crystals grown in 200mM NaCl | | pH4 + 100mM NaCl  crystals grown in 200mM NaCl | |
| --- | --- | --- | --- | --- | --- | --- | --- | --- | --- |
| Unit cell dimensions | | a= 81.5 Å, b= 103.2 Å, γ=90° | | a= 81.6 Å, b= 104.2 Å, γ=90° | | a= 82 Å, b= 104 Å, γ=90° | | a= 81 Å, b= 104 Å, γ=90° | |
| Space group | | *p*22_1_2_1_ | | | | | | | |
| Resolution | | 6 Å | | | | | | | |
| No. of images | | 9 | | 11 | | 9 | | 5 | |
| Range of defocus | | 287-747 nm | | 433-750 nm | | 311-1324 nm | | 594-813 nm | |
| Total no. of reflections^a^ | | 1523 | | 2028 | | 1724 | | 917 | |
| No. of unique reflections^b^ | | 192 | | 198 | | 199 | | 178 | |
| Overall phase residual to 6 Å  (AVRGAMPS, random= 90°)^a^ | | 33.7° | | 30.5° | | 30.5° | | 29.7° | |
| Overall phase residual to 6 Å  (sym. constraint, random= 45°)^b^ | | 23.6° | | 20.8° | | 19.9° | | 20.4° | |
| Statistics  (FOMSTATS,  random= 45°) | Res (Å)  19.2  13.5  11.0  9.5  8.5  7.8  7.2  6.7  6.3  6.0 | Reflections  21  20  21  18  20  17  20  20  16  19 | PhRes (°)  12.0  14.4  19.1  16.2  15.5  18.4  31.0  32.6  36.2  43.9 | Reflections  21  20  22  18  21  20  19  18  17  22 | PhRes (°)  17.6  12.7  18.5  23.1  11.2  23.4  14.8  16.2  36.5  35.2 | Reflections  23  20  21  18  20  20  21  18  16  22 | PhRes (°)  11.9  10.2  12.7  17.6  13.1  19.4  22.1  34.7  26.9  33.1 | Reflections  18  21  22  16  20  18  21  17  15  15 | PhRes (°)  13.9  10.4  22.4  14.2  15.4  18.1  24.9  23.4  24.4  40.9 |

^a^ Calculated from the program AVRGAMPS: mean weighted squared distance of the phase values from the averaged value (90° = random) ^b^ Calculated from the program FOMSTATS: averaged phase values from the symmetry-constrained target values of 0° or 180° (45° = random). ^a,b^ Reflections with IQ ≤ 7 Å were included.

**Supplementary file 1: Electron crystallographic data. (continued)**

| Condition | | pH8 -NaCl | | pH8 + 20 mM NaCl | | pH8 + 50 mM NaCl | | pH8 + 100 mM NaCl | |
| --- | --- | --- | --- | --- | --- | --- | --- | --- | --- |
| Unit cell dimensions | | a= 81.2 Å, b= 104.2 Å, γ=90° | | a= 80.7 Å, b= 104.6Å, γ=90° | | a= 80.5 Å, b= 106.1 Å, γ=90° | | a= 80.9 Å, b= 106.3 Å, γ=90° | |
| Space group | | *p*22_1_2_1_ | | | | | | | |
| Resolution | | 6 Å | | | | | | | |
| No. of images | | 15 | | 10 | | 11 | | 7 | |
| Range of defocus | | 595-971 nm | | 322-652 nm | | 331-728 nm | | 473-872 nm | |
| Total no. of reflections^a^ | | 2858 | | 1894 | | 1689 | | 1409 | |
| No. of unique reflections^b^ | | 194 | | 198 | | 196 | | 199 | |
| Overall phase residual to 6 Å  (AVRGAMPS, random= 90°)^a^ | | 29.2° | | 32.2° | | 30.4° | | 29.7° | |
| Overall phase residual to 6 Å  (sym. constraint, random= 45°)^b^ | | 16.2° | | 21.9° | | 21.1° | | 18.4° | |
| Statistics  (FOMSTATS,  random= 45°) | Res (Å)  19.2  13.5  11.0  9.5  8.5  7.8  7.2  6.7  6.3  6.0 | Reflections  18  20  22  18  21  19  20  19  17  20 | PhRes (°)  12.5  14.1  15.1  16.6  14.3  13.5  11.7  23.9  20.8  20.6 | Reflections  21  20  23  17  21  19  21  18  18  20 | PhRes (°)  26.8  14.2  14.4  19.0  10.9  23.8  26.5  33.2  29.1  23.9 | Reflections  21  20  22  19  19  20  19  18  19  19 | PhRes (°)  13.2  6.2  10.8  15.6  11.2  19.6  27.4  31.1  39.1  41.0 | Reflections  23  18  22  21  18  21  18  18  19  21 | PhRes (°)  17.1  8.5  16.6  10.2  9.3  14.2  16.7  32.3  22.2  36.7 |

^a^ Calculated from the program AVRGAMPS: mean weighted squared distance of the phase values from the averaged value (90° = random) ^b^ Calculated from the program FOMSTATS: averaged phase values from the symmetry-constrained target values of 0° or 180° (45° = random). ^a,b^ Reflections with IQ ≤ 7 Å were included.

**Supplementary file 1: Electron crystallographic data. (continued)**

| Condition | | pH8 + 250 mM NaCl | | pH8 + 500 mM NaCl | | pH8 + 20 mM LiCl | | pH8 + 100 mM LiCl | |
| --- | --- | --- | --- | --- | --- | --- | --- | --- | --- |
| Unit cell dimensions | | a= 79.8 Å, b= 106.4 Å, γ=90° | | a= 80.6 Å, b= 108 Å, γ=90° | | a= 80.4 Å, b= 104.3 Å, γ=90° | | a= 81.2Å, b= 104.75 Å, γ=90° | |
| Space group | | *p*22_1_2_1_ | | | | | | | |
| Resolution | | 6 Å | | | | | | | |
| No. of images | | 6 | | 12 | | 9 | | 2 | |
| Range of defocus | | 304-704 nm | | 305-753 nm | | 331-751 nm | | 540-550 nm | |
| Total no. of reflections^a^ | | 1200 | | 2644 | | 1580 | | 352 | |
| No. of unique reflections^b^ | | 190 | | 204 | | 194 | | 161 | |
| Overall phase residual to 6 Å  (AVRGAMPS, random= 90°)^a^ | | 34.6° | | 26.2° | | 30.7° | | 31.1° | |
| Overall phase residual to 6 Å  (sym. constraint, random= 45°)^b^ | | 21° | | 15.9° | | 22.6° | | 27.1° | |
| Statistics  (FOMSTATS,  random= 45°) | Res (Å)  19.2  13.5  11.0  9.5  8.5  7.8  7.2  6.7  6.3  6.0 | Reflections  19  18  23  20  19  19  18  17  18  19 | PhRes (°)  15.2  9.7  16.7  13.1  11.1  30.2  29.5  30.8  26.0  30.3 | Reflections  22  21  21  21  18  20  22  19  20  20 | PhRes (°)  19.1  12.5  15.6  9.4  10.7  11.8  15.2  17.0  19.7  28.3 | Reflections  21  20  21  17  21  18  21  18  19  18 | PhRes (°)  19.1  11.1  14.8  21.5  23.4  18.0  28.8  34.3  28.7  27.8 | Reflections  19  19  19  19  18  17  14  7  15  14 | PhRes (°)  16.3  11.5  25.5  23.4  24.6  34.0  44.5  33.0  28.8  42.4 |

^a^ Calculated from the program AVRGAMPS: mean weighted squared distance of the phase values from the averaged value (90° = random) ^b^ Calculated from the program FOMSTATS: averaged phase values from the symmetry-constrained target values of 0° or 180° (45° = random). ^a,b^ Reflections with IQ ≤ 7 Å were included.

**Supplementary file 1: Electron crystallographic data. (continued)**

| Condition | | pH8 + 100 mM KCl | | pH8 + 10 mM MgCl_2_ | | pH8 - NaCl  crystals grown in 200mM NaCl | | pH8 + 100mM NaCl  crystals grown in 200mM NaCl | |
| --- | --- | --- | --- | --- | --- | --- | --- | --- | --- |
| Unit cell dimensions | | a= 81.9 Å, b= 104 Å, γ=90° | | a= 81.8 Å, b= 103.8 Å, γ=90° | | a= 81.8 Å, b= 104.5 Å, γ=90° | | a= 81.1 Å, b= 104 Å, γ=90° | |
| Space group | | *p*22_1_2_1_ | | | | | | | |
| Resolution | | 6 Å | | | | | | | |
| No. of images | | 7 | | 6 | | 4 | | 4 | |
| Range of defocus | | 401-598 nm | | 484-717 nm | | 496-638 nm | | 270-637 nm | |
| Total no. of reflections^a^ | | 1434 | | 1016 | | 800 | | 667 | |
| No. of unique reflections^b^ | | 194 | | 187 | | 195 | | 180 | |
| Overall phase residual to 6 Å  (AVRGAMPS, random= 90°)^a^ | | 25.5° | | 31.4° | | 31.2° | | 35.2° | |
| Overall phase residual to 6 Å  (sym. constraint, random= 45°)^b^ | | 19° | | 30.9° | | 30.5° | | 20° | |
| Statistics  (FOMSTATS,  random= 45°) | Res (Å)  19.2  13.5  11.0  9.5  8.5  7.8  7.2  6.7  6.3  6.0 | Reflections  19  18  22  18  20  21  19  17  17  23 | PhRes (°)  8.1  8.5  14.3  19.2  13.0  14.8  22.9  27.1  20.7  39.0 | Reflections  20  20  21  19  20  17  18  17  16  19 | PhRes (°)  15.4  20.1  29.4  23.1  25.4  33.3  36.7  45.5  49.8  37.0 | Reflections  23  20  20  18  19  20  20  17  17  21 | PhRes (°)  28.5  21.6  21.2  23.5  25.7  38.7  33.4  32.4  45.9  35.8 | Reflections  18  20  22  17  21  17  20  15  16  14 | PhRes (°)  17.0  11.1  8.6  15.3  12.0  19.5  33.2  38.6  19.6  34.7 |

^a^ Calculated from the program AVRGAMPS: mean weighted squared distance of the phase values from the averaged value (90° = random) ^b^ Calculated from the program FOMSTATS: averaged phase values from the symmetry-constrained target values of 0° or 180° (45° = random). ^a,b^ Reflections with IQ ≤ 7 Å were included.

**Supplementary file 1: Electron crystallographic data. (continued)**

| Condition | | pH6 -NaCl | | pH6 + 250 mM NaCl | | pH6 + 500 mM NaCl | | pH6 + 1 M NaCl | |
| --- | --- | --- | --- | --- | --- | --- | --- | --- | --- |
| Unit cell dimensions | | a= 81.5 Å, b= 103.6 Å, γ=90° | | a= 80.9 Å, b= 103.5 Å, γ=90° | | a= 81.3 Å, b= 103.3 Å, γ=90° | | a= 80.7 Å, b= 104.1 Å, γ=90° | |
| Space group | | *p*22_1_2_1_ | | | | | | | |
| Resolution | | 6 Å | | | | | | | |
| No. of images | | 9 | | 17 | | 11 | | 7 | |
| Range of defocus | | 431-738 nm | | 221-703 nm | | 303-466 nm | | 447-662 nm | |
| Total no. of reflections^a^ | | 1619 | | 2956 | | 2008 | | 1146 | |
| No. of unique reflections^b^ | | 196 | | 197 | | 196 | | 195 | |
| Overall phase residual to 6 Å  (AVRGAMPS, random= 90°)^a^ | | 30.6° | | 32.9° | | 26.3° | | 31° | |
| Overall phase residual to 6 Å  (sym. constraint, random= 45°)^b^ | | 21.9° | | 21.7° | | 18.3° | | 24.1° | |
| Statistics  (FOMSTATS,  random= 45°) | Res (Å)  19.2  13.5  11.0  9.5  8.5  7.8  7.2  6.7  6.3  6.0 | Reflections  22  20  22  17  21  19  18  20  17  20 | PhRes (°)  16.7  11.2  15.5  17.2  16.0  24.8  28.7  25.5  37.7  30.0 | Reflections  22  21  21  17  22  17  21  19  18  19 | PhRes (°)  15.6  9.0  11.8  12.4  15.3  19.7  24.7  44.5  38.0  29.7 | Reflections  21  20  21  18  21  18  20  20  17  20 | PhRes (°)  14.8  9.6  8.9  10.9  24.3  16.0  17.6  26.6  23.0  31.6 | Reflections  22  20  23  17  20  19  22  18  16  18 | PhRes (°)  18.9  11.5  15.9  15.4  14.2  14.5  25.7  53.6  32.5  45.1 |

^a^ Calculated from the program AVRGAMPS: mean weighted squared distance of the phase values from the averaged value (90° = random) ^b^ Calculated from the program FOMSTATS: averaged phase values from the symmetry-constrained target values of 0° or 180° (45° = random). ^a,b^ Reflections with IQ ≤ 7 Å were included.

**Supplementary file 1: Electron crystallographic data. (continued)**

| Condition | | pH6 + 500 mM LiCl | |  | |  | |  | |
| --- | --- | --- | --- | --- | --- | --- | --- | --- | --- |
| Unit cell dimensions | | a= 80.6 Å, b= 104 Å, γ=90° | |  | |  | |  | |
| Space group | | *p*22_1_2_1_ | |  | |  | |  | |
| Resolution | | 6 Å | |  | |  | |  | |
| No. of images | | 6 | |  | |  | |  | |
| Range of defocus | | 398-621 nm | |  | |  | |  | |
| Total no. of reflections^a^ | | 1033 | |  | |  | |  | |
| No. of unique reflections^b^ | | 185 | |  | |  | |  | |
| Overall phase residual to 6 Å  (AVRGAMPS, random= 90°)^a^ | | 35.7° | |  | |  | |  | |
| Overall phase residual to 6 Å  (sym. constraint, random= 45°)^b^ | | 22.7° | |  | |  | |  | |
| Statistics  (FOMSTATS,  random= 45°) | Res (Å)  19.2  13.5  11.0  9.5  8.5  7.8  7.2  6.7  6.3  6.0 | Reflections  19  19  22  17  19  18  21  16  16  18 | PhRes (°)  18.9  9.0  13.9  26.0  14.4  24.1  23.1  34.2  36.6  33.5 |  |  |  |  |  |  |

^a^ Calculated from the program AVRGAMPS: mean weighted squared distance of the phase values from the averaged value (90° = random) ^b^ Calculated from the program FOMSTATS: averaged phase values from the symmetry-constrained target values of 0° or 180° (45° = random). ^a,b^ Reflections with IQ ≤ 7 Å were included.
